# Virocell resource manipulation under nutrient limitation

**DOI:** 10.1128/msystems.00521-25

**Published:** 2025-06-24

**Authors:** Morgan M. Lindback, Cristina Howard-Varona, Jane Fudyma, Malak Tfaily, Matthew B. Sullivan, Melissa B. Duhaime

**Affiliations:** 1 Department of Ecology and Evolutionary Biology, University of Michigan 1259, Ann Arbor, Michigan, USA; 2 Department of Microbiology, The Ohio State University 2647, Columbus, Ohio, USA; 3 Department of Environmental Science, University of Arizona 8041, Tucson, Arizona, USA; 4 Department of Civil, Environmental and Geodetic Engineering, The Ohio State University 2647, Columbus, Ohio, USA; 5 Center for RNA Biology, The Ohio State University 2647, Columbus, Ohio, USA; Third Institute of Oceanography Ministry of Natural Resources, Xiamen, China

**Keywords:** phage, microbe, virocells, environment, multi-omics

## Abstract

Virus-infected cells, called virocells, impact host metabolic functions, resources, and ecosystem processes, but the effects of nutrient limitation remain less well understood. Here, we leverage transcriptomic, proteomic, and endo- and exo-metabolomic data from two *Pseudoalteromonas* virocells independently infected by unrelated dsDNA viruses, PSA-HS2 (HS2-virocells) and PSA-HP1 (HP1-virocells), to examine how phosphate limitation affects virocell resource manipulation intra- and extracellularly. Intracellularly, we find that (i) HP1-virocells boost amino acid production toward the end of the infection cycle but deplete amino acid pools relative to HS2-virocells; (ii) both virocells dampen the production of *de novo* nucleotide synthesis proteins; (iii) HS2-virocells switch from *de novo* synthesis to recycling of phospholipids, whereas HP1-virocells decrease both activities; (iv) all cells (virocells and uninfected cells), but HP1-virocells especially, increase membrane fluidity; and (v) both virocells increase iron storage. Extracellularly, (i) polyphenols, a stress marker, increased in all cells, particularly in HP1-virocells, and (ii) only HP1-virocells showed elevated unsaturated hydrocarbons and oxygen-rich metabolites, which are likely byproducts of intracellular metabolic activity. These findings advance our understanding of how environmental conditions shape virocell activities in ecologically relevant nutrient-limited conditions and reveal distinct responses of virocells to infection by unrelated viruses.

**IMPORTANCE:**

This study addresses a knowledge gap in understanding how nutrient limitation shapes virus-infected bacterial cell (virocell) metabolism and its ecosystem footprints. Using multi-omics approaches, we examined how two different viruses (PSA-HP1 and PSA-HS2) independently infecting the same marine heterotrophic bacterium (*Pseudoalteromonas*) respond to phosphorus limitation. Building upon our previous work, we show how virocell metabolic reprogramming manipulates cellular resources and alters the extracellular environment. Intracellularly, while both virocells reprogram similar metabolic pathways, they manipulate key resources (nucleotides, amino acids, lipids, and iron) distinctly under nutrient limitation. Extracellularly, each virocell generates unique dissolved organic matter metabolites, with a differential expression of stress markers under phosphorus limitation, indicating environment-specific ecosystem footprints. These results provide fundamental insights into how virocell metabolic reprogramming and resource manipulation combine to produce ecosystem-scale metabolic outputs.

## INTRODUCTION

Marine microbes are crucial drivers of biogeochemical cycling (1–3), with an estimated 20–40% of surface ocean microbes being virus-infected and 10^23^ viral infections occurring every second (1, 4, 5). Virus-infected cells—virocells—are genetically, phenotypically, and physiologically distinct from uninfected cells (6), as virocell metabolism is reprogrammed to replicate the virus rather than the host (7, 8). Most studies of virocell metabolic reprogramming focus on “central” metabolic pathways (e.g., carbon, nitrogen, energy), which are virally reprogrammed either through viral-encoded auxiliary metabolic genes (AMGs) (9–14) or, in systems lacking AMGs, through regulators and global cellular changes (15–18). This extensive body of research has revealed that virocells alter many bacterial metabolic pathways, including electron flow and photosynthesis (13, 19–21), defense systems (16, 22), translation and energy (17, 18, 23, 24), amino acid synthesis (24–28), nucleotide synthesis (24, 28–37), and lipid metabolism (18, 26, 38–41), with recent global ocean studies suggesting many viral metabolic functions remain to be discovered (14).

Beyond their intracellular effects, virocell metabolic reprogramming significantly impacts the extracellular environment and ecosystem processes. From the few available studies, we have learned that (i) virocells source nitrogen from the environment for building proteins (27), (ii) uninfected cells benefit from the labile lysate obtained during infection (24, 42), (iii) intracellular metabolites produced by virocells can be released to benefit other organisms (13, 43), and (iv) nutrient limitation dictates how cells and virocells transform extracellular organic matter (18). These studies exemplify the importance of studying connections between virocell metabolic reprogramming and environmental impacts, especially under less-well-characterized conditions, such as nutrient limitation, for which laboratory model systems can be beneficial.

Our previous work in a marine virocell model system has contributed to the understanding of both intracellular and extracellular impacts of virocells across environmental conditions. Previously, we infected *Pseudoalteromonas marina* 13-15 independently with dsDNA phages HS2 (a 38 Kb dsDNA siphovirus) or HP1 (a 45 Kb dsDNA podovirus) under high- (17) or low-phosphate (*P*) (18) conditions, where the high-P medium had 11 and 77× more organic and inorganic *P* (P_i_), respectively, than the low-P medium (18). Using comprehensive multi-omics measurements (transcriptomics, proteomics, endometabolomics, exometabolomics, and lipidomics), we found that metabolic reprogramming in high-P conditions was phage-specific and hypothesized that the increased metabolic reprogramming by phage HP1 stemmed from the codon mismatch between that phage and the host (17). We then compared the metabolic reprogramming strategies between high- and low-P conditions and found the environment was a strong driver of virocell metabolic reprogramming and altered the virocells ecosystem footprints (18). Both virocells showed a canonical P-limitation response and gained resources for lytic infections by assimilating nitrogen (N), rerouting central carbon metabolism, and degrading fatty acids (18). Low-P virocells also transformed less organic matter in the environment and selectively consumed P-rich lipids and N-rich peptides (18). The results of these studies pointed toward virocell resource manipulation for lytic infection and prompted us to further examine how virocells gain resources and influence their environment.

Here, we again leverage data from our marine virocell model system to examine in detail how P-limitation affects resource manipulation and its impact on extracellular metabolites, two critical aspects for understanding virocell ecology under nutrient limitation.

## MATERIALS AND METHODS

### Bacterial growth and phage infections

Growth and infections of *Pseudoalteromonas marina* 13-15 were studied under phosphate-limited conditions using high- and low-P media comprising 1% Zobell with specific nutrient additions to achieve high- and low-P treatments. Additional details, including growth curves and infections, are included for reference (Fig. S1 and S2). The high-P medium contained 55 µM total organic phosphate (TOP) and 48 µM inorganic phosphate (PO_4_), while the low-P medium had 5 µM TOP and 0.6 µM PO_4_.

### Transcriptomics, proteomics, lipidomics, endometabolomics, and exometabolomics

Transcriptomics was conducted by collecting and pelleting diluted samples from HP1- and HS2-virocells at various time points, followed by flash-freezing and RNA extraction. The RNA quality was verified, and sequencing was performed using HiSeq2500. Raw gene counts were generated and normalized, with differential expression analyses using edgeR. Metabolic pathways were reconstructed with Kyoto Encyclopedia of Genes and Genomes (44) and EcoCyc (45).

For proteomics, 80 mL was collected in triplicates from diluted samples at various time points, pelleted, flash-frozen, and proteins extracted. Mass spectrometry analysis using a Q-Exactive Plus identified peptides with MS-GF+ and specific search parameters. Data are available at MassIVE (MSV000083626) and ProteomeXchange (PXD013204).

For lipidomics, 40–80 mL of diluted samples was collected, pelleted, washed with PBS, and flash-frozen. For endometabolomics, 80–90 mL of diluted samples was vacuum-filtered, washed with PBS, and stored until extraction. MPLEx extraction was used for intracellular lipids and endometabolites. Lipid identification was performed using LIQUID. Endometabolite MS data were analyzed using a metabolite detector, matched to metabolomics databases, and validated manually. Peak area values were log-transformed for further analyses. Three media-only blanks were included.

For exometabolites, 90 mL of diluted samples was collected in triplicates, spun down to pellet cells, filtered, and flash-frozen. Three media-only blanks were included. After salt removal using solid-phase extraction (46), high-resolution mass spectra were obtained using a Bruker 9.4-Tesla FT-ICR MS. Chemical formulas were assigned with Formultitude software (previously named Formularity [47]).

### Linear mixed-effects models

Separate linear mixed-effects models were fitted for various data sets, including endo-amino acids, lipidomics, and exometabolomic data, incorporating a random intercept to account for gene correlations. Main effects, interactions, and their significance in predicting counts were assessed with Type II Wald *χ*-test analysis of variance to determine the variance explained by each term.

### Interpretation of data and results

Our multi-omics analysis revealed distinct metabolic responses to phosphate limitation across uninfected and virus-infected cells. Below, we first define the key terminology used throughout this section, then examine the impacts of phosphate limitation on intracellular resource manipulation and extracellular metabolites.

**Fig 1:**
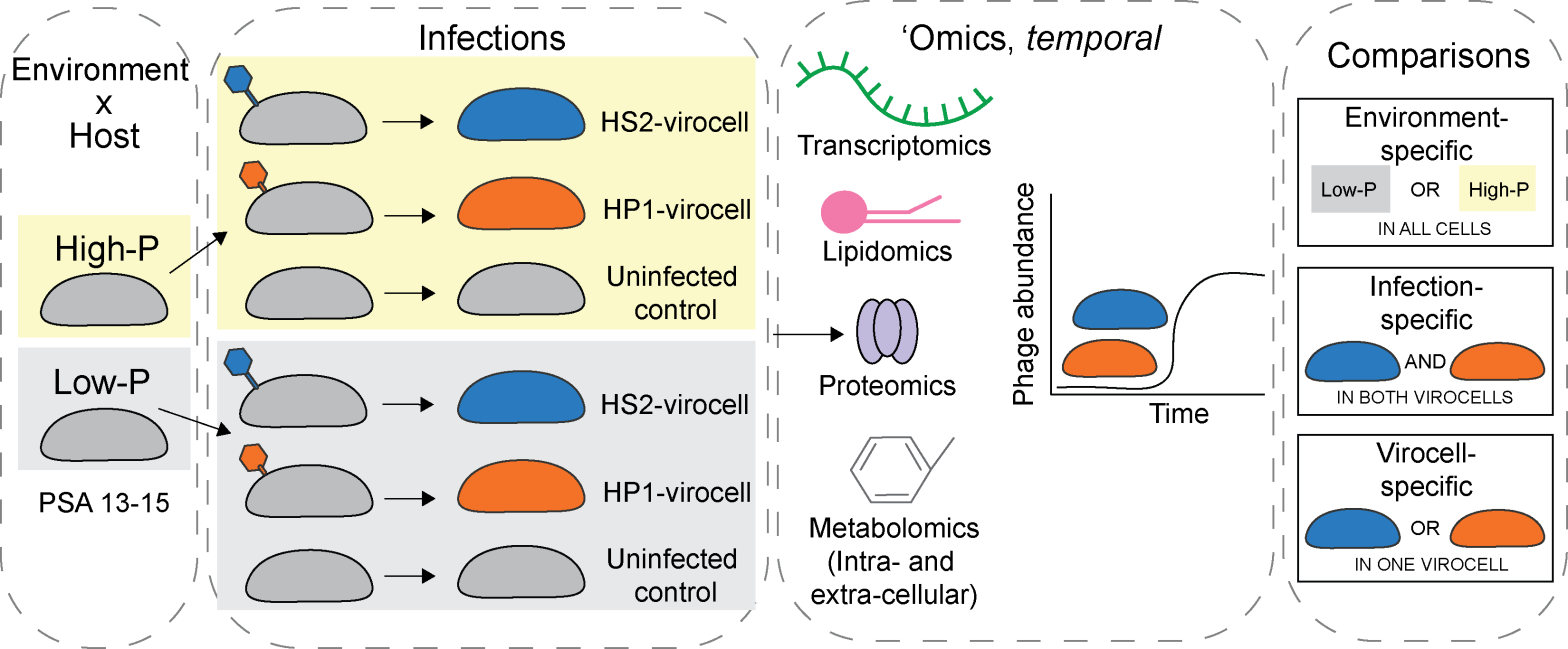
Experimental design. *Pseudoalteromonas marina* sp. 13-15 was independently infected with phages PSA-HS2 or PSA-HP1 generating HS2- and HP1-virocells, respectively, under high- (high-P) or low-phosphate (low-P) environments. Temporally resolved samples were collected for transcriptomics, proteomics, endo-lipidomics, endo-metabolomics, and exo-metabolomics. This work discusses (i) environment-specific effects (i.e., those that are shared in all cells and virocells due to the changing environment), (ii) infection-specific effects (i.e., those that are shared between both virocells, but not uninfected cells, as a result of infection in either environment), and (iii) virocell-specific effects (i.e., those that are unique to a single virocell due to the infecting phage).

Throughout the results, we use standardized terminology to describe various treatments and outcomes outlined here for clarity. Overall, we use the terms environment-, infection-, and virocell-specific to describe, respectively, effects on (i) all cells (uninfected cells and both virocells) equally in response to low-P, (ii) both virocells equally, but not uninfected cells, in response to phage infection, and (iii) each virocell differently in response to the specific infecting phage (Fig. 1). Biomolecule terminology is as follows: transcripts are described as being over-expressed (OE; also “over-expression”) (fold-change > 1), under-expressed (UE; also “under-expression”) (fold-change < 1), or not differentially expressed (not DE; fold-change = 1) in infected relative to uninfected cells in the same condition and time point. Proteins are described as enriched (*z*-score across all proteins, timepoints, treatments, and replicates > 0), depleted (*z*-score < 0), or not different from the mean (z-score = 0). Lipids and metabolites are described as enriched (fold-change > 1) or depleted (fold-change < 1) in infected relative to uninfected cells in the same condition and time point. All instances of ‘Time’ are considered time post-infection (such that *T*0 is 15 min after phage have been added, which allows time for absorption to the host based on prior knowledge of this phage-host system) (17). Host growth and infection curves (Fig. S1 and S2) are included for reference.

## RESULTS AND DISCUSSION

### Intracellular virocell impacts of low-P

#### Low-P HP1-virocells boost amino acid synthesis machinery more than HS2-virocells but deplete free amino acids

During infection, cellular N and P are rerouted toward the synthesis of viral nucleotides and proteins (27, 48). We previously found (17) that both HP1- and HS2-virocells manipulated central carbon, N, and P metabolisms, which are all critical pathways for resource synthesis. Building upon these findings, we investigated how these pathway alterations affected amino acid and nucleotide synthesis.

**Fig 2:**
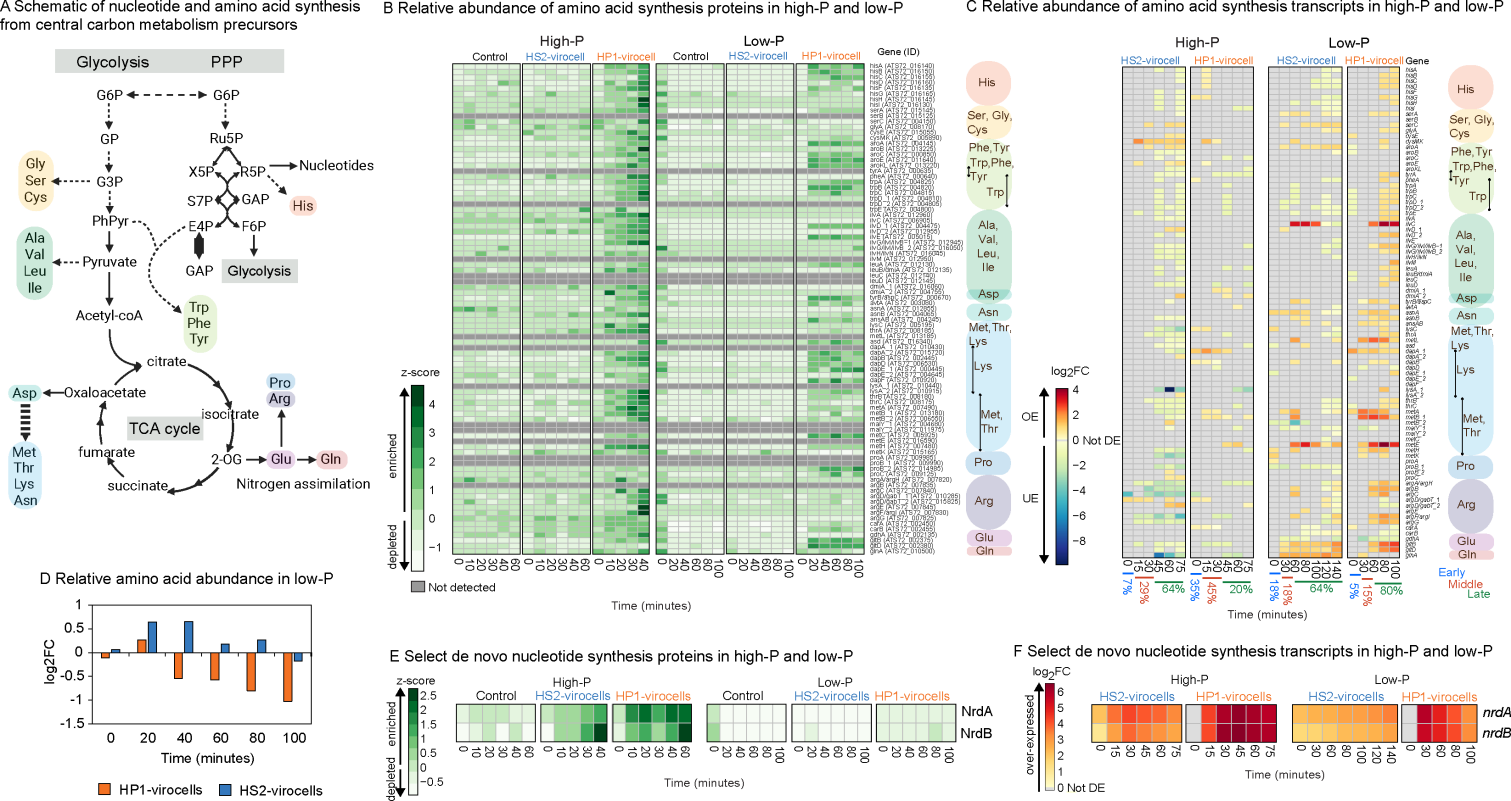
Amino acid and nucleotide resource production in all cells. (**A**) Schematic representation of central carbon metabolism pathways and the precursors generated from these pathways that produce amino acids through nitrogen assimilation and nucleotides *de novo*. (**B**) Heatmap of relative protein abundance of the amino acid synthesis proteins in high- and low-P conditions for uninfected control cells and both virocells obtained from proteomics. Represented are *z*-scores. Proteins are considered enriched if *z*-score > 0 and depleted if *z*-score < 0, and dark gray is a protein not detected in our data set. (**C**) Heatmap of expression of the amino acid synthesis genes in the high- and low-P virocells relative to uninfected control cells in each respective condition, obtained from transcriptomics. Gray denotes genes that are not differentially expressed (not DE). “OE” is over-expressed, while “UE” is under-expressed. (**D**) Relative abundance of all amino acids detected in the endometabolome represented as log_2_FC in the virocells relative to uninfected control cells in the same environment (low-P) and time point; accompanying linear mixed effects model and statistics are available in Table S1. (**E**) Heatmap of expression of the ribonucleoside-diphosphate reductase (*nrd*) genes in the high-. and low-P virocells relative to uninfected control cells in each respective condition, obtained from transcriptomics. (**F**) Heatmap of relative protein abundance of Nrd proteins in high- and low-P conditions for uninfected control cells and both virocells, obtained from proteomics. Represented are *z*-scores. Proteins are considered enriched if *z*-score > 0 and depleted if *z*-score < 0.

Our analysis of 85 host amino acid synthesis genes revealed distinct strategies between the two virocells. For proteins, HP1-virocells enriched a greater fraction (≥55%) of those genes than both uninfected cells and HS2-virocells (≤42%), regardless of the environment (Fig. 2B; Fig. S3A). For transcripts, HP1-virocells OE a higher fraction (approximately twofold) of those same genes than HS2-virocells relative to uninfected cells in both environments (Fig. 2C; Fig. S3B). Additionally, ≥80% of those transcripts were OE during late infection stages (≥60 min) in low-P only (Fig. 2C). These data suggest that HP1-virocells increase amino acid synthesis more than HS2-virocells in both environments, especially toward the end of the infection cycle in low-P.

If new amino acid production occurs late in the infection for HP1-virocells in response to low-P (as our evidence suggested), we would expect virocells to recycle existing host amino acids early during infection. We term this ‘*the amino acid source switch hypothesis*’ whereby, in response to the changing environment, HP1-virocells switch amino acid sources from recycling to synthesis as infection progresses. A different “source switch” was previously observed in cyanovirocells where N for phage amino acids is derived from the host early in the infection and switched to environment acquisition late in the infection (27). Seeing another switch here for HP1-virocells, but not for HS2-virocells, suggests a virocell-specific capability of switching resource acquisition based on availability and speaks to the distinct infection needs of viruses for replication under different environmental regimes (17, 18).

We next leveraged endometabolome amino acid measurements to assess how their relative abundances changed across infections and environments using a previously developed linear mixed-effects model (18). This revealed that in low-P, HS2-virocells had significantly higher amino acid abundances than the uninfected control (*P* value < 0.05; Table S1), whereas HP1-virocells had significantly lower amino acid abundances than both HS2-virocells and the uninfected control (*P* value < 0.05; Table S1), with a significant decrease over time (*P* value < 0.05; Fig. 2D; Table S1). The opposite occurred in high-P: HS2-virocells had significantly lower amino acid abundances than the uninfected control (*P* value < 0.05; Table S1), while HP1-virocells had significantly higher amino acid abundances than HS2-virocells (*P* value < 0.05; Table S1) but were not significantly different from the uninfected control (*P* value > 0.05; Table S1).

These results, coupled with transcript and protein data, suggest environment-, infection-, and virocell-specific effects on amino acid pools. In high-P, HP1-virocells boosted amino acid synthesis and were able to maintain an amino acid surplus relative to HS2-virocells by “working harder” (17). Here, we reveal that in low-P conditions, HP1-virocells initially recycle host amino acids early in the infection but switch to synthesizing new amino acids during late infection. Despite this increased synthesis, amino acid abundances are lower in HP1-virocells than in HS2-virocells. We hypothesize that this discrepancy occurs because either the demand for amino acids exceeds their production (e.g., due to high protein synthesis) or HP1-virocells have less efficient amino acid synthesis than HS2-virocells. We previously found that codons encoded by phage HS2 are more similar to host *Pseudoalteromonas marina* sp. 13-15 than those of phage HP1, and we previously hypothesized that this mismatch in available resources (amino acids and tRNAs) between the host and HP1 may lead to the metabolic reprogramming strategies and lower fitness we observed between the two virocells in high-P media (17). Our data here suggest that this mismatch in resources between HP1 and the host may be exacerbated in low-P, leading to the need to synthesize new amino acids late in infection. In summary, amino acid resource manipulation is environment-, infection-, and virocell-specific, and it occurs through a “source switch.”

With the “source switch” hypothesis, we suggest that phage first rely on host resources for amino acids before synthesizing or importing. This builds upon previous work that showed infection can increase amino acid availability by sourcing from extracellular N, especially later in infection (27). However, that study did not investigate more than one virocell. Others have shown high variability between phages in their intracellular free amino acid concentrations (33), suggesting that amino acid manipulation is phage-specific, but lacked the comparison between environments. By comparing two virocells under two different *P* regimes, we were able to observe and compare virocell resource manipulation strategies across environments and build upon previous observations to show (i) that all infected cells, regardless of environment, enhance amino acid synthesis (17, 18), and (ii) virocells may employ different metabolic reprogramming strategies with measurable consequences (e.g., HS2-virocells have more free amino acids than HP1). These examples together now show that cellular and environmental quotas for resources dictate how building blocks are sourced for replication in a virocell-specific manner.

#### Virocells transcriptionally upregulate *de novo* nucleotide synthesis, but protein abundance is dampened in low-P

Ribonucleoside-diphosphate reductase (*nrd*) genes, which are conserved in all organisms, catalyze the rate-limiting step in *de novo* nucleotide production and are thus important during phage infection, as they ensure the supply of essential deoxyribonucleotides needed for phage genome replication (14, 37, 49–53). While they are commonly found in phage genomes (14), they are not encoded by PSA-HS2 or PSA-HP1. We previously found that the PSA host *nrd* genes had both the highest and longest temporal expression during infection in both virocells in high-P (17). Similarly, in low-P, *nrdA* and *nrdB* were among the genes with highest and longest OE in both virocells (Fig. 2F; Fig. S3D). The proteins, however, which we did not previously examine, were only enriched in high-P and depleted in low-P for both virocells (Fig. 2E; Fig. S3E). Overall, these findings suggest environment- and infection-specific effects on nucleotide synthesis transcripts and proteins. Environment-specific effects result in both virocells limiting Nrd protein production in low-P relative to high-P conditions. Infection-specific effects result in both virocells OE *nrd* genes, regardless of the environment. Because of the high *nrd* transcription, regardless of the environment, these genes may be transcriptional markers of HP1 and HS2 infection on this host, as has been similarly proposed in other systems (54, 55).

#### Low-P alters virocell phospholipid synthesis strategies

**Fig 3:**
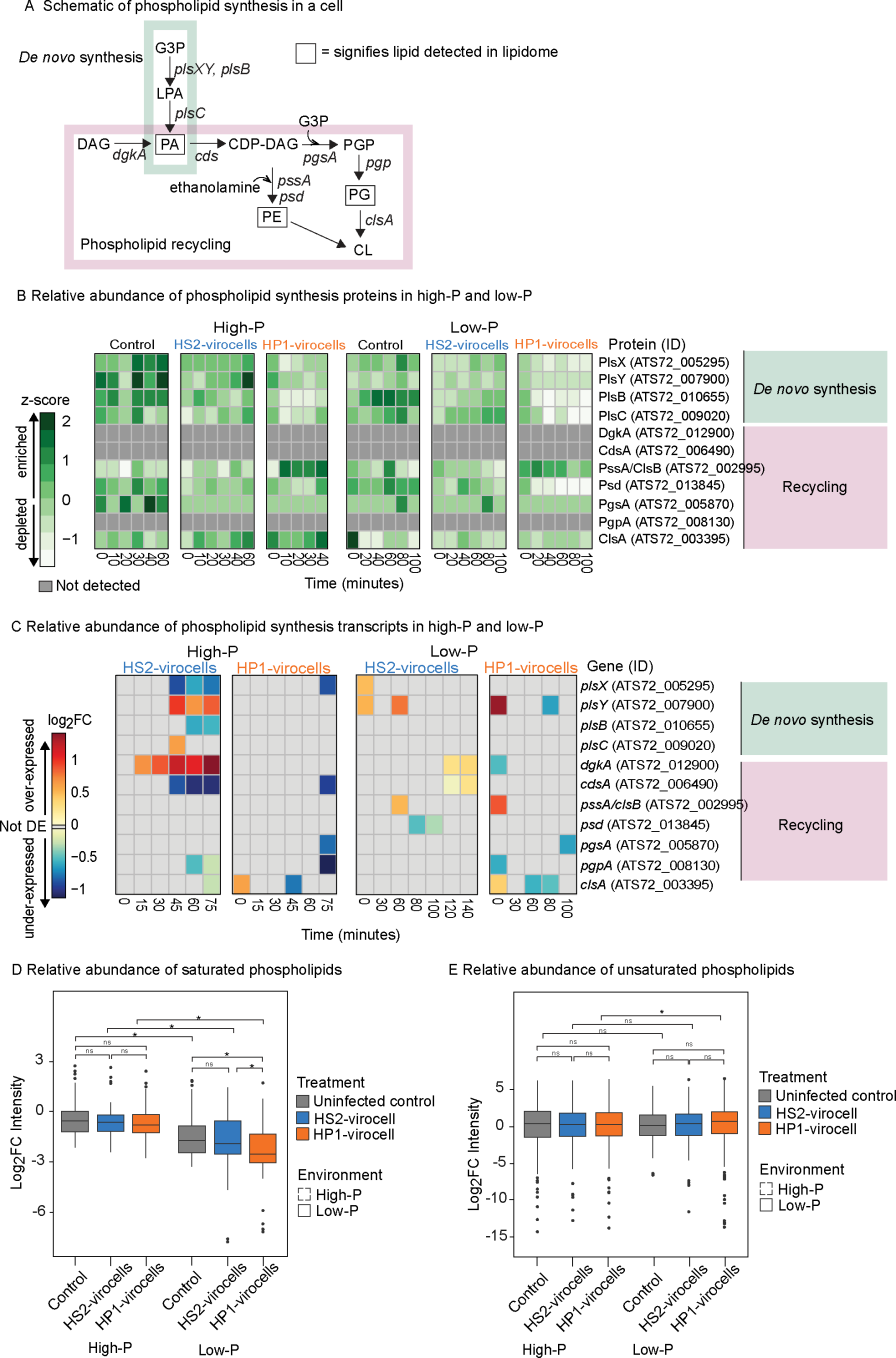
Phospholipid synthesis in all cells. (**A**) Cartoon summarizing phospholipid synthesis *de novo* and phospholipid modification in a cell. Acronyms: G3P, glycerol-3-phosphate; LPA, lysophosphatidic acid; PA, phosphatidic acid; DAG, diacylglycerol; CDP-DAG, cytidine diphosphate-diacylglycerol; PE, phosphatidylethanolamine; PGP, phosphatidylglycerol phosphate; PG, phosphatidylglycerol; CL, cardiolipin; OE, overexpressed; UE, underexpressed; DE, differentially expressed; PlsX, phosphate acyltransferase; PlsY, acyl phosphate:glycerol-3-phosphate acyltransferase; PlsB, glycerol-3-phosphate 1-O-acyltransferase B; DgkA, diacylglycerol kinase A; CdsA, CDP-diglyceride synthetase A; PssA, phosphatidylserine synthase A; PgsA, CDP-diacylglycerol-glycerol-3-phosphate 3-phosphatidyltransferase A; PgpA, phosphatidylglycerophosphatase A; ClsA, cardiolipin synthase A. (**B**) Heatmap of the relative protein abundance of the phospholipid synthesis and recycling genes in both high- and low-P conditions for uninfected control cells and both virocells, obtained from proteomics. Represented are *z*-scores. Proteins are considered enriched if *z*-score > 0 and depleted if *z*-score < 0. Dark gray denotes a protein not detected in our data set. (**C**) Heatmap of the expression of the phospholipid synthesis and recycling genes in both high- and low-P conditions for the virocells relative to uninfected control cells in each respective condition and time point, obtained from transcriptomics. Light gray denotes genes that are not differentially expressed (not DE). “OE” is over-expressed (log_2_FC > 0); “UE” is under-expressed (log_2_FC < 0). (**D**) Relative abundance of saturated phospholipids (*n* = 9) from biological triplicates of the lipidomics data. (**E**) Relative abundance of unsaturated phospholipids (*n* = 70) from biological triplicates of the lipidomics data. For both D and E, statistics are shown from the marginal analysis of variance of the linear mixed-effects model. The *P* values indicate if a variable significantly affects the response variable after controlling for other variables. Degrees-of-freedom method: Kenward-Roger; *P* value adjustment: Tukey’s method for comparing a family of three estimates. “*” denotes significant comparisons (*P* value < 0.05), and “ns” denotes “not significant” comparisons (*P* value ≥ 0.05). Additional statistics on these data can be found in Tables S2 and S3.

Building on our previous observation that HP1-virocells had fewer total lipids than HS2-virocells in low-P (18), we investigated specific changes in phospholipid metabolism—a key cellular response to phosphate stress in marine bacteria (56). Metabolically, phospholipids can be synthesized either *de novo* from central carbon metabolism intermediate glycerol-3-phosphate (herein ‘*de novo* phospholipid synthesis’), which makes glycerophosphates, or by modifying existing glycerophosphates into other classes of phospholipids (herein ‘phospholipid recycling’), including glycerophosphoethanolamines and glycerophospholipids (57) (Fig. 3A).

**Fig 4:**
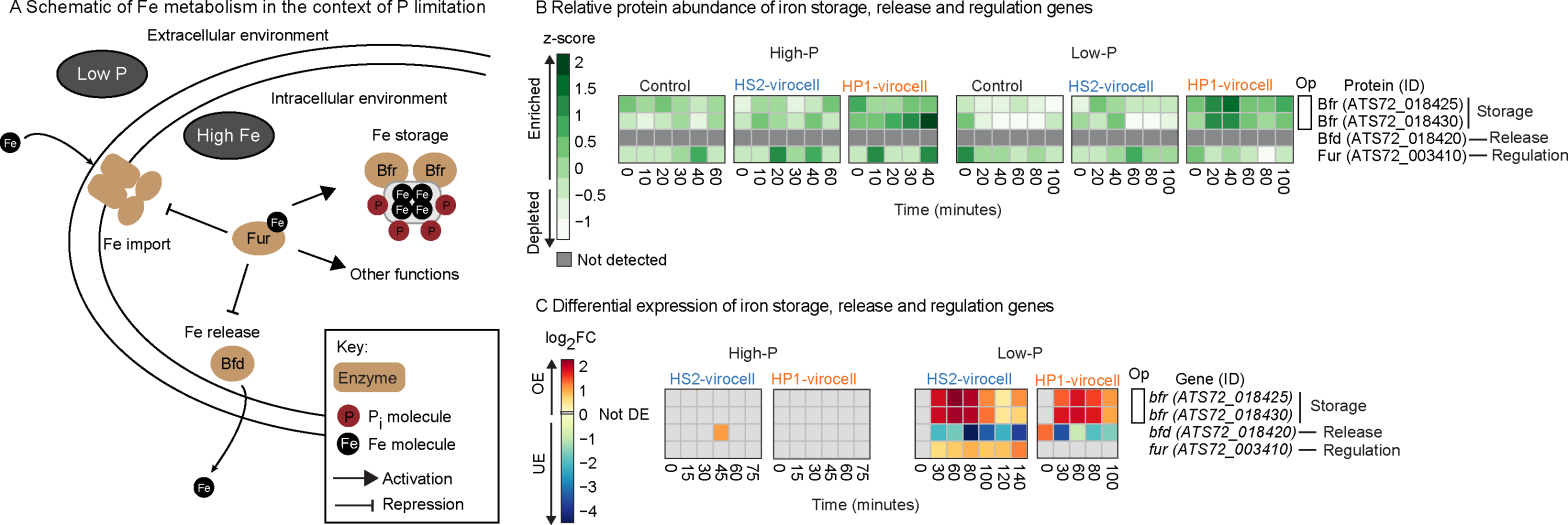
Iron (Fe) storage, release, and regulation in all cells. (**A**) Cartoon representation of iron (Fe) metabolism in a cell in a low-P environment depicting the regulation of iron storage and release by Fur and the stoichiometry between Fe and *P* storage. Shown is that for every molecule of stored Fe; one of *P* is also stored. (**B**) Heatmap of the relative protein abundance (*z*-score across all samples and environment) in high- and low-P environments, obtained from proteomics. Proteins are considered enriched if their *z*-score > 0 and depleted if their *z*-score < 0. (**C**) Heatmap with relative gene expression (log_2_FC relative to uninfected control cells in the same environment and time point), obtained from transcriptomics. Genes are OE if log_2_FC > 0, UE if log_2_FC < 0, and not DE if log_2_FC = 0. The gene, ID, and function are the same for both heatmaps. Represented are the Fe metabolism genes (storage, release, and regulation by Fur).

Our analysis revealed that while uninfected cells maintained consistent phospholipid synthesis strategies across both environments, virocells showed environment-specific adaptations. In uninfected cells, the same fraction of proteins involved in phospholipid *de novo* synthesis (100%; Fig. S4A) and recycling (57%; Fig. 4C) was enriched in both environments (Fig. S4A). However, low-P led to virocell-specific changes in phospholipid synthesis strategies, such that HS2-virocells switched from *de novo* synthesis to recycling, and HP1-virocells decreased both modes, as follows: in HS2-virocells, the fraction of enriched proteins involved in *de novo* phospholipid synthesis was 100% in high-P and 75% in low-P (Fig. 3B; Fig. S4A), with 50% of those genes OE in both environments (Fig. 3C; Fig. S4B). In contrast, the fraction of enriched proteins involved in phospholipid recycling was 43% in high-P and 57% in low-P, with the fraction of OE genes involved in phospholipid recycling increasing from 14% in high-P to 43% in low-P (Fig. 3C; Fig. S4D). HP1-virocells reduced the fraction of enriched proteins in response to low-P for both *de novo* synthesis (75% in high-P and 50% in low-P; Fig. 3B; Fig. S4A) and recycling (43% in high-P and 29% in low-P; Fig. 3C; Fig. S4C). Additionally, fewer genes were OE in low-P compared to HS2-virocells for both *de novo* synthesis (25% vs 50%; Fig. 3C; Fig. S4B) and recycling (14% vs 43%; Fig. 3C; Fig. S4D).

While changes in membrane remodeling and phospholipid composition are common responses to P-limitation in bacteria (56), there is little knowledge about how those strategies manifest in virocells. We found that uninfected *Pseudoalteromonas* cells do not alter their phospholipid acquisition strategies under different *P* conditions. Recent work has suggested that bacteria may face an ecological tradeoff when undergoing lipid remodeling, as they may become more susceptible to grazing by predators (57). In contrast, virocells do adjust their strategies and in virocell-specific ways, invoking either *de novo* phospholipid synthesis or recycling based on P availability.

#### Low-P cells, especially HP1-virocells, increase membrane fluidity

Since membrane phospholipids are known to change in response to both the environment (58) and viral infection (59–62), and considering the observed environment- and virocell-specific effects on phospholipid synthesis strategies, we next leveraged the phospholipid data to infer how low-P and infection impacted host membrane fluidity. First, for environment-specific effects, we found that all cells (uninfected and both virocells) had significantly fewer saturated phospholipids in low-P than in high-P (*P* value < 0.05; Table S2; Fig. 3D), but unsaturated phospholipids did not significantly change between environments for any of the cells (*P* value > 0.05; Table S3; Fig. 3E). Second, for virocell-specific effects in low-P, HS2-virocells did not significantly differ in the abundance of saturated phospholipids relative to uninfected cells (*P* value > 0.05, Table S2; Fig. 3D), whereas HP1-virocells had significantly fewer saturated phospholipids than uninfected cells (*P* value < 0.05; Table S2; Fig. 3D). Since saturated phospholipids create rigid membranes by packing more tightly together, these results suggest that (i) low-P led to increased membrane fluidity by reducing the amount of saturated phospholipids in all cells (environment-specific effect); (ii) infection did not result in a shared response between the two virocells; and (iii) HP1-virocells may have the most fluid membranes (virocell-specific effect).

Although the lipidome is understudied in response to viral infection, our results support current ideas in the field that membrane composition and fluidity change in response to (i) the environment and (ii) infection and (iii) that these responses may be phage-specific. In a one-host two-prophage *Sulfitobacter* virocell system, both nutrients and prophage infection significantly impacted the lipidome, with nutrients being a significantly stronger driver in lipidome response (61). Changes to the lipidome, especially fluidity, are known to affect the viral life cycle in other systems. For example, T4 infection is known to alter *Escherichia coli* phospholipid composition (63), which authors suggest may have direct or indirect impacts on virus fitness. Such changes to lipid composition and fluidity could indirectly impact phage fitness through mechanisms, such as co-infection dynamics; for example, DNA injection of phage PhiX174 is hindered by a more rigid membrane (64). More directly, phage may have higher fitness with the ability to alter phospholipid composition, such as T4 (48, 65). Together, our results and the literature suggest that changes to phospholipid membrane composition and fluidity are highly influenced by the environment and may impact viral fitness in a virus-specific manner.

#### Low-P virocells store iron

Iron (Fe) storage proteins (bacterioferritins) associate with P at ~1Fe:1P ratios such that storing Fe results in storing P (66). This is ecologically important since Fe is a trace element in the oceans (67) that co-limits with P (68–72). Bacteria may regulate both metabolisms through global regulators, *pho* for Pi and *fur* for iron, which use metabolic crosstalk to adjust their metabolism to environmental conditions (73). Given this, we hypothesized that low-P would increase Fe storage and decrease Fe release in our virocell systems. To test this hypothesis, we examined transcripts and proteins for bacterioferritin (*bfr*) and bacterioferritin-associated ferredoxin (*bfd*) genes involved in Fe storage and release, respectively, as well as for the ferric uptake regulator protein (*fur*) that controls Fe uptake, storage, and release (74, 75) (Fig. 4A).

In both environments and in all cells (virocells and uninfected), Fur (Fe regulation) and Bfr (Fe storage) proteins were enriched, and Bfd (Fe release) was not detected (Fig. 4B; Fig. S5). This suggests that Fe metabolism proteins are unaffected by infection or environment. Transcriptionally, however, there was a difference between environments. Both virocells continuously OE *bfr* and UE *bfd* from mid to late infection (>=30 min). However, they did so only in low-P, as neither *bfr* nor *bfd* was DE in high-P. These data suggest environment- and infection-specific effects whereby low-P enhances Fe storage and represses its release in both virocells, potentially storing the same amount of P. Additionally, there were virocell-specific effects by which low-P HS2-virocells co-*expressed fur* and *bfr* (Fig. 4C; Fig. S5). We interpreted this co-expression as a signal that low-P HS2-virocells direct Fe metabolism through Fur. These findings suggest that Fe storage and release are another example of phage HS2’s ability to more finely tune ancillary host metabolism, similar to its previously reported control over the canonical P_i_-stress response and nitrogen assimilation (18).

Phage are already known to take advantage of iron as a trace element via the “Ferrojan Horse Hypothesis” (76), in which the tails of some marine phages are critical keepers of Fe and trick bacteria into obtaining such Fe, leading to infection. This Fe could then be recycled during infection to build virions, but how virocells manipulate Fe metabolism during infection is less known, especially across infections and varying nutrient regimes. Our data suggest (i) an infection-specific effect in response to low-P, whereby both virocells are greater keepers of Fe when in low-P, and (ii) a virocell-specific effect, whereby HS2-virocells orchestrate Fe metabolism and its ramifications for other cellular functions through the global regulator *fur*. This work builds on the existing literature to show that the Fe evolutionary arms race between viruses and their hosts may go beyond viral structures to include metabolic reprogramming strategies and opens the door to investigating how viruses may take advantage of their hosts’ global regulators.

### Extracellular metabolites provide new insights into virocell ecosystem footprints in low-P

While intracellular metabolic changes are well documented during viral infection, the impact of nutrient limitation on virocell-mediated transformation of extracellular metabolites remains poorly understood. It is already known that cells and virocells change metabolite consumption and production in response to varying nutrient conditions and infection, as they transform organic matter for resources (12, 17, 18, 24, 27, 42, 77, 78) and produce metabolites that signal other cells (43, 79). Previously, we found that phosphate availability significantly influenced the overall exometabolome composition in this system (18). Here, we specifically examined how phosphate limitation affects the production of secondary metabolites during infection—compounds known to be produced by *Pseudoalteromonas* species (80, 81) with roles in cell-cell communication or responses to phage infection (33, 79) but rarely studied under varying nutrient conditions and viral infection.

**Fig 5:**
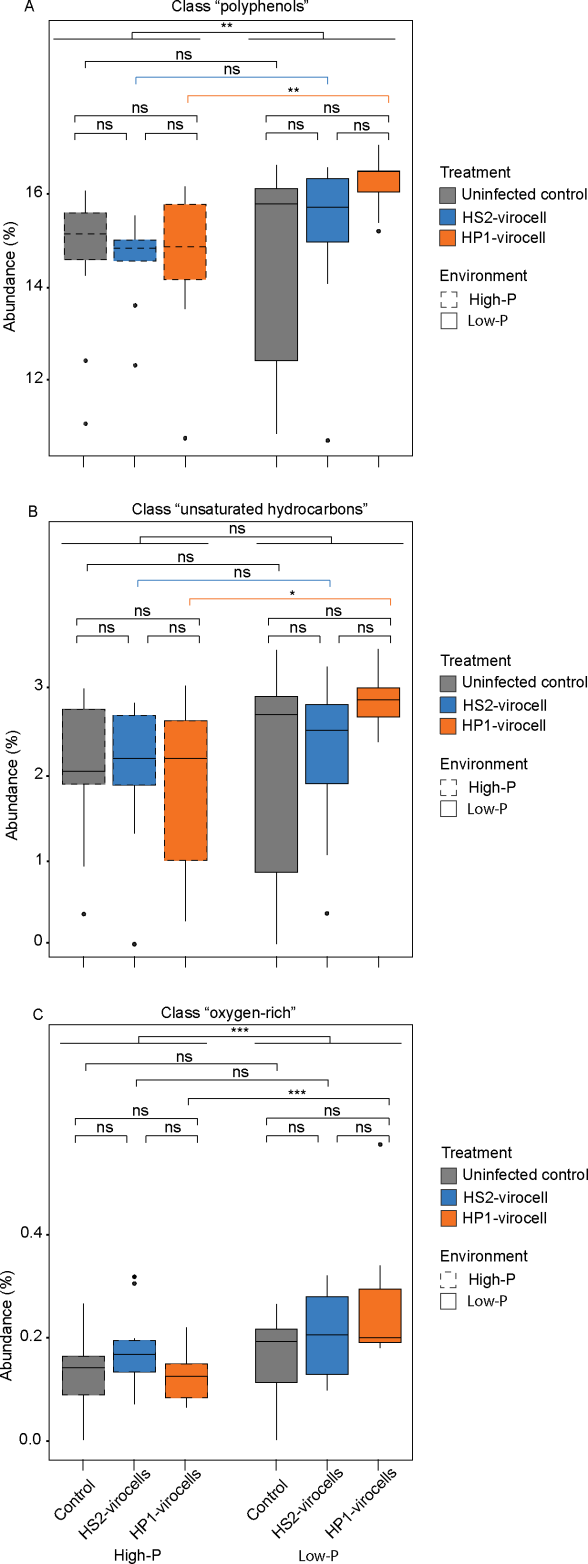
Ecosystem footprint inferred from select exometabolites. Boxplots representing the range of percentages of total exometabolites identified in each treatment that were classified as (**A**) polyphenol-like, (**B**) unsaturated hydrocarbon-like, and (**C**) oxygen-rich metabolites for uninfected cells and HS2- and HP1-virocells in both environments for biological quadruplicates. Significance tests from lowest to highest horizontal lines denote the *P* value resulting from a Wilcoxon test comparing virocells to uninfected cells, virocells to each other, or between high- and low-P environments by cell type or all cell types grouped together, with “*” denoting significant comparisons (*P* value < 0.05) and “ns” denoting “not significant” comparisons (*P* value ≥ 0.05).

Our analysis focused on three key classes of exometabolites associated with microbial dissolved organic matter (82): polyphenol-like, unsaturated hydrocarbons-like, and oxygen-rich metabolites (O/C ratio ~0.5) that significantly changed in response to environmental conditions and/or phage infection prior to lysis. Across all cells, the extracellular environment was enriched in polyphenols and oxygen-rich metabolites (Wilcox, *P-adj* value < 0.05; Fig. 5A through C). Viral infection by phage HP1 triggered distinct responses under phosphate limitation, while uninfected cells and HS2-virocells maintained consistent levels of these compounds across environments (Wilcox, *P-adj* value > 0.05; Fig. 5). Specifically, HP1-virocells significantly increased all three metabolite classes (polyphenol-like, unsaturated hydrocarbons-like, and oxygen-rich metabolites) in low-P conditions (Wilcox, *P-adj* value < 0.05; Fig. 5A through C) but not in high-P (Wilcox, *P-adj* value > 0.05; Fig. 5A through C). These phage-specific responses suggest a distinct response to the combined stresses of HP1 infection under phosphate stress. First, the elevated polyphenol production, particularly in HP1-virocells, likely represents a stress response to infection under nutrient limitation. This aligns with the known roles of polyphenols in marine organisms as mediators of cell-cell communication and stress responses (83), including in *Pseudoalteromonas* species (76). Second, the increased hydrocarbon release by low-P HP1-virocells correlates with their observed intracellular lipid depletion (18), potentially indicating enhanced lipid degradation under stress (84–87). Third, the elevated oxygen-rich metabolites in low-P HP1-virocells mirror similar increases observed in virus-infected cyanobacteria (88) and virus-infected algae (89), suggesting a phage-specific infection response across diverse marine systems.

The differential production of these metabolites has important ecological implications. Both hydrocarbons and oxygen-rich metabolites can serve as energy and carbon sources for other microbes (85, 90–92), suggesting that HP1-virocells may differently influence the surrounding microbial community through their distinct metabolite profiles. These findings highlight how nutrient limitation can shape virus-host interactions beyond the cellular level, leading to virus-specific impacts on marine dissolved organic matter pools and microbial community dynamics.

### Conclusions

While it has been observed (77) and modeled (93) that viruses reprogram their hosts to gain resources needed for replication, the specifics of how viral metabolic reprogramming changes between hosts (11, 16, 22), viruses (15, 17, 18), communities (13, 43), and environments (12, 18, 94) is only now coming to light. It is now clear that viruses encode metabolic genes (9, 95) that likely manipulate more than one-third of marine microbial metabolic pathways known in global ocean data sets (14). However, the nuances of virocell metabolic reprogramming–particularly where the virus genomes lack such metabolism-targeting genes–remain understudied. This knowledge is critical for establishing the ecosystem impacts of virus infection and establishing ‘virocell’ states that could be represented in ecosystem and biogeochemical models.

This study experimentally determines the intra- and extracellular consequences of nutrient limitation on the resource manipulation of two different virocells derived from independently infecting the same host with two viruses. There are infection- and virocell-specific effects observed both intracellularly and extracellularly, which illuminates the challenge the field will have in extrapolating from the existing measurements of a limited number of experimental phage-host systems. Effectively capturing viruses in molecular- to Earth system-scale models will require a field-wide effort that expands the diversity of phage-host systems and nutrient conditions studied. It will also require a transition from focus on lytic infections to more broadly represent data across the ‘virus infection continuum’ (96), including non-virulent chronic (97) or lysogenic (16, 98) infections. Finally, extracellular biomolecules are critical for understanding microbial system connectivity, as they operate as both communication and currency for microbial communities (99) and thus serve as connections between intracellular metabolism, microbial interactions, and environmental impacts of viral infection. However, current untargeted metabolomics approaches need improvement in measurement, annotation, and analysis capabilities. While a daunting task, a field-wide effort to collectively advance understanding of virocell metabolic reprogramming and ecosystem impacts is critical to the cross-scale modeling needed to bring virocells, along with microbes, into climate models (100, 101). As “puppet masters of the marine microbial realm” (76), viruses and their hosts must be accurately represented to predict ecosystem-scale responses to environmental change.

## Data Availability

Data are available at Zenodo (https://zenodo.org/records/14548518) and Cyverse (http://datacommons.cyverse.org/browse/iplant/home/shared/iVirus/Pseudoalteromonas_Omics). This paper leverages data and experiments from previous work, where the methods are explained in great detail (18). While no new experiments were performed here, a summary of the methods used in that previous work is presented for convenience. Data are available at Zenodo (https://zenodo.org/records/10355633) for both the previous publication (18), from which the raw data were obtained to perform the analyses presented here, and an updated version (v3) with the new analyses from this paper. Scripts are also available at Cyverse (http://datacommons.cyverse.org/browse/iplant/home/shared/iVirus/Pseudoalteromonas_Omics). All genomes are available at the Joint Genome Institute’s portal for Integrated Microbial Genomes/Virus (IMG/VR) and GenBank. The IMG submission IDs are 100976 (*Pseudoalteromonas marina* 13-15, 4.09 Mbp; https://img.jgi.doe.gov/cgi-bin/mer/main.cgi?section=TaxonDetail&page=taxonDetail&taxon_oid=2703719062), 44764 (PSA-HS2 37.72 Kbp dsDNA; https://img.jgi.doe.gov/cgi-bin/vrer/main.cgi?section=TaxonDetail&page=taxonDetail&taxon_oid=2582581232), and 279897 (PSA-HP1, 45.06 Kbp dsDNA; https://img.jgi.doe.gov/cgi-bin/vrer/main.cgi?section=TaxonDetail&page=taxonDetail&taxon_oid=2974666577). The GenBank IDs are FSRF00000000 (*Pseudoalteromonas marina* 13-15), KF302036.1 (PSA-HS2), and NC_048630 (PSA-HP1). All cultures are available through the National Collection of Industrial, Food and Marine Bacteria (NCIMB) collection. The NCIMB IDs are NCIMB15513 (*Pseudoalteromonas marina* 13-15), NCIMB15514 (PSA-HP1), and NCIMB15515 (PSA-HS2). The genome of *Pseudoalteromonas marina* strain 13-15 was downloaded from NCBI (http://ncbi.nlm.nih.gov) on 20 March 2018.

## SUPPLEMENTAL MATERIAL

Supplemental Material msystems.00521-25-s0001.docx Supplemental figures and tables.

ASM does not own the copyrights to Supplemental Material that may be linked to, or accessed through, an article. The authors have granted ASM a non-exclusive, world-wide license to publish the Supplemental Material files. Please contact the corresponding author directly for reuse.
