# Supplementary material for: Virocell resource manipulation under nutrient limitation
Source: mSystems. 2025 Jun 24;10(7):e00521-25. doi: 10.1128/msystems.00521-25 (PMC12282109; doi:10.1128/msystems.00521-25)
Supplement: Supplemental Material — Supplemental figures and tables. [file msystems.00521-25-s0001.docx]

**SUPPLEMENTARY INFORMATION**

# **Contents**

1. **Figure S1.** Bacterial host growth in varying phosphate (P) conditions.
2. **Figure S2.** Infection dynamics of phage PSA-HS2 in both media.
3. **Figure S3.** Amino acid and nucleotide synthesis.
4. **Figure S4.** Phospholipid synthesis.
5. **Figure S5**. Iron metabolism proteins.
6. **Table S1.** Results from the linear mixed effects model of amino acids from intracellular metabolomics data between conditions and infection types at the final time point.
7. **Table S2.** Results from the linear mixed effects model of the saturated lipids in the lipidomics data (mean-centered, log_2_-fold change of the intensity values).
8. **Table S3.** Results from the linear mixed effects model of the unsaturated lipids in the lipidomics data (mean-centered, log2-fold change of the intensity values).
9. **Table S4.** Results from the linear mixed effects model of the unsaturated lipids in the lipidomics data (mean-centered, log2-fold change of the intensity values).


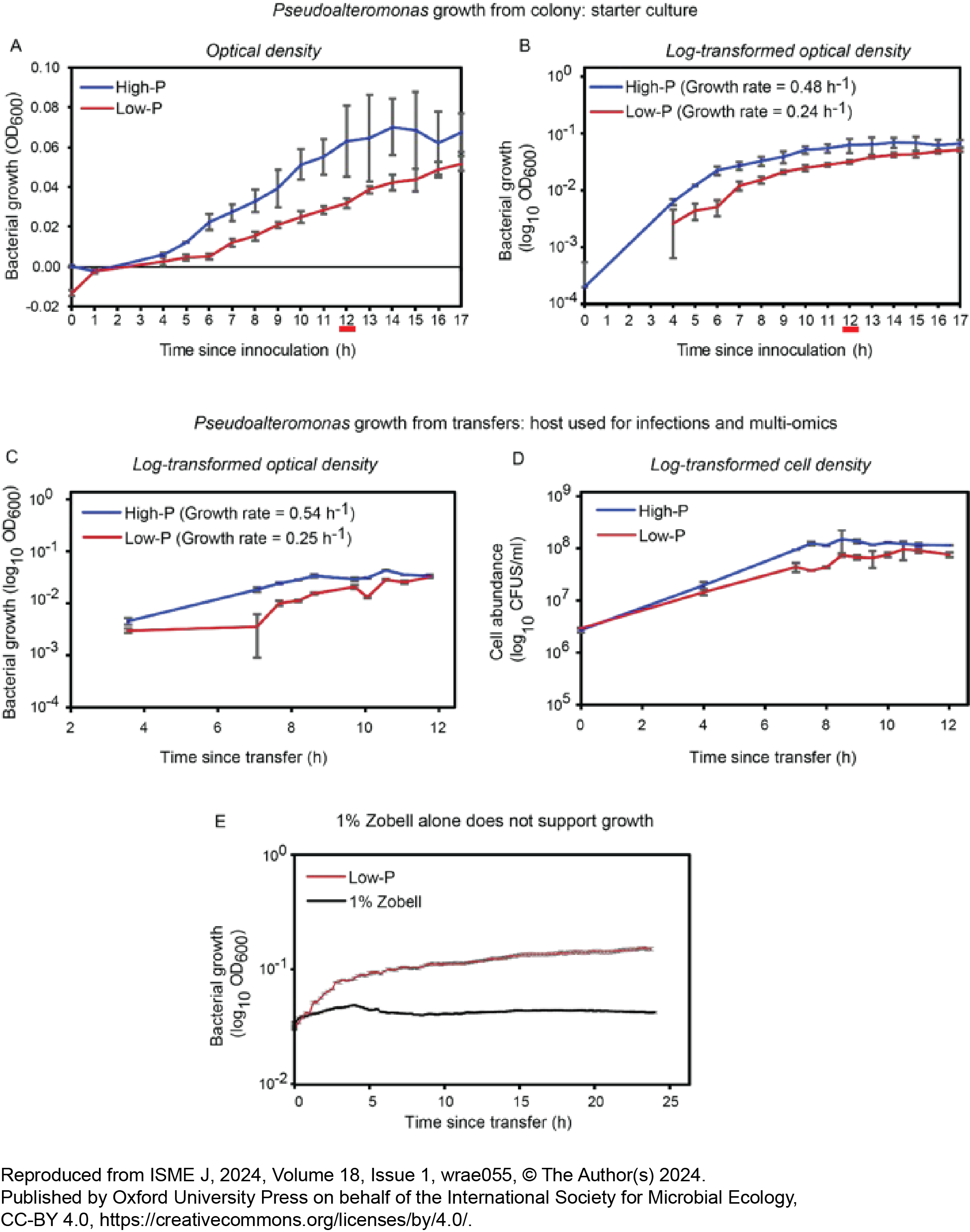


**Figure S1** Bacterial host growth in varying phosphate (P) conditions. *Pseudoalteromonas sp*. 13-15 growth from a colony into 10 mL of a high-P (1% Zobell + CNP) or a low-P (1% Zobell + CN) medium presented as raw optical density (OD) values (A) or log_10_-transformed (B). This represented the starter culture which, after 12 hours (as indicated by the red line), was transferred into 200 ml of the same medium (C, D) and enabled to grow until reaching ~10^8^ cells/ml to begin phage infections. Both log_10_-transformed OD values (C) and cell density (cfus/ml, D) were obtained for the transfer cultures. Growth rates are represented for both cultures (B, C). Cell growth was also tested in 1% Zobell with no added C, N, or P sources alongside the low-P medium (E), inoculated first from a colony as in A-B, then transferred as in C-D which is what is represented. For all plots, medium-only blank values were subtracted to the OD values of the growth treatments, and the average of 2-3 biological replicates and their standard error are plotted.


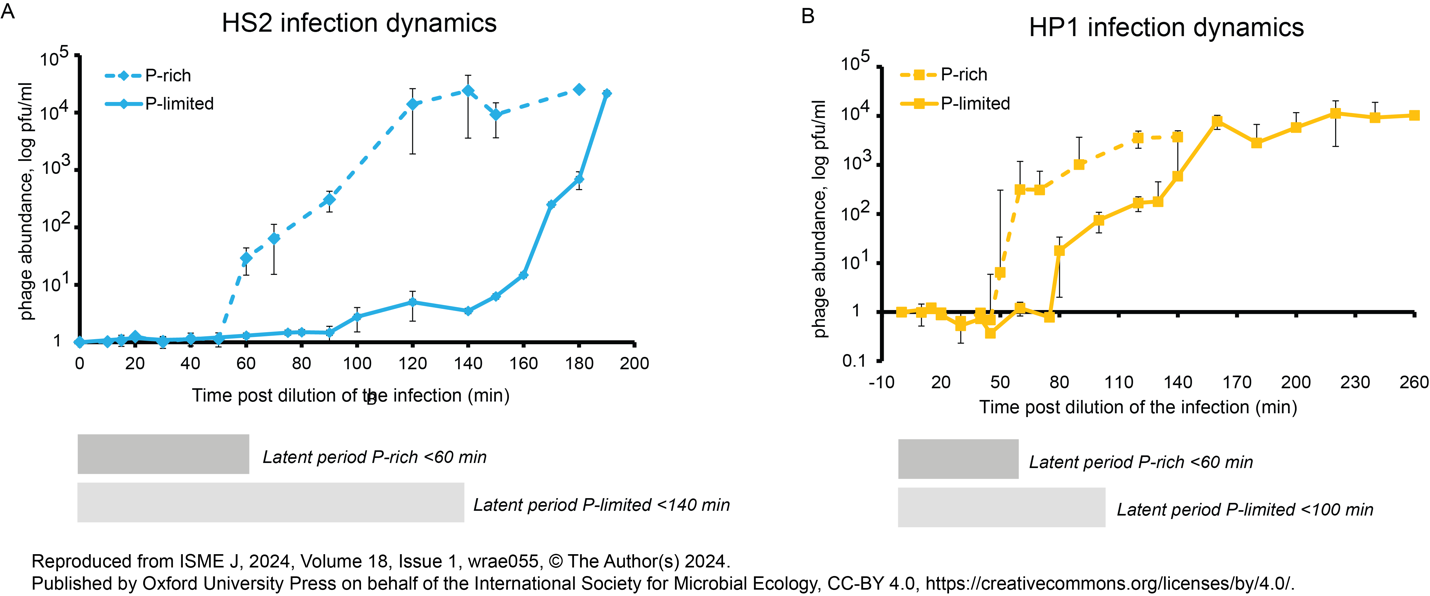


**Figure S2:** (**A**) Infection dynamics of phage PSA-HS2 in both media. (**B**) Infection dynamics of phage PSA-HP1 in both media. For both, (A) and (B), phage abundance, measured as plaque-forming units (pfus) per mL for free phages are represented over time, and 0 min represents 15 min after diluting the infection. The average of three biological replicates is plotted along with the error. All data points have been normalized against the first time point (0 min). Estimates of latent period are represented with rectangle boxes under each graph.

##
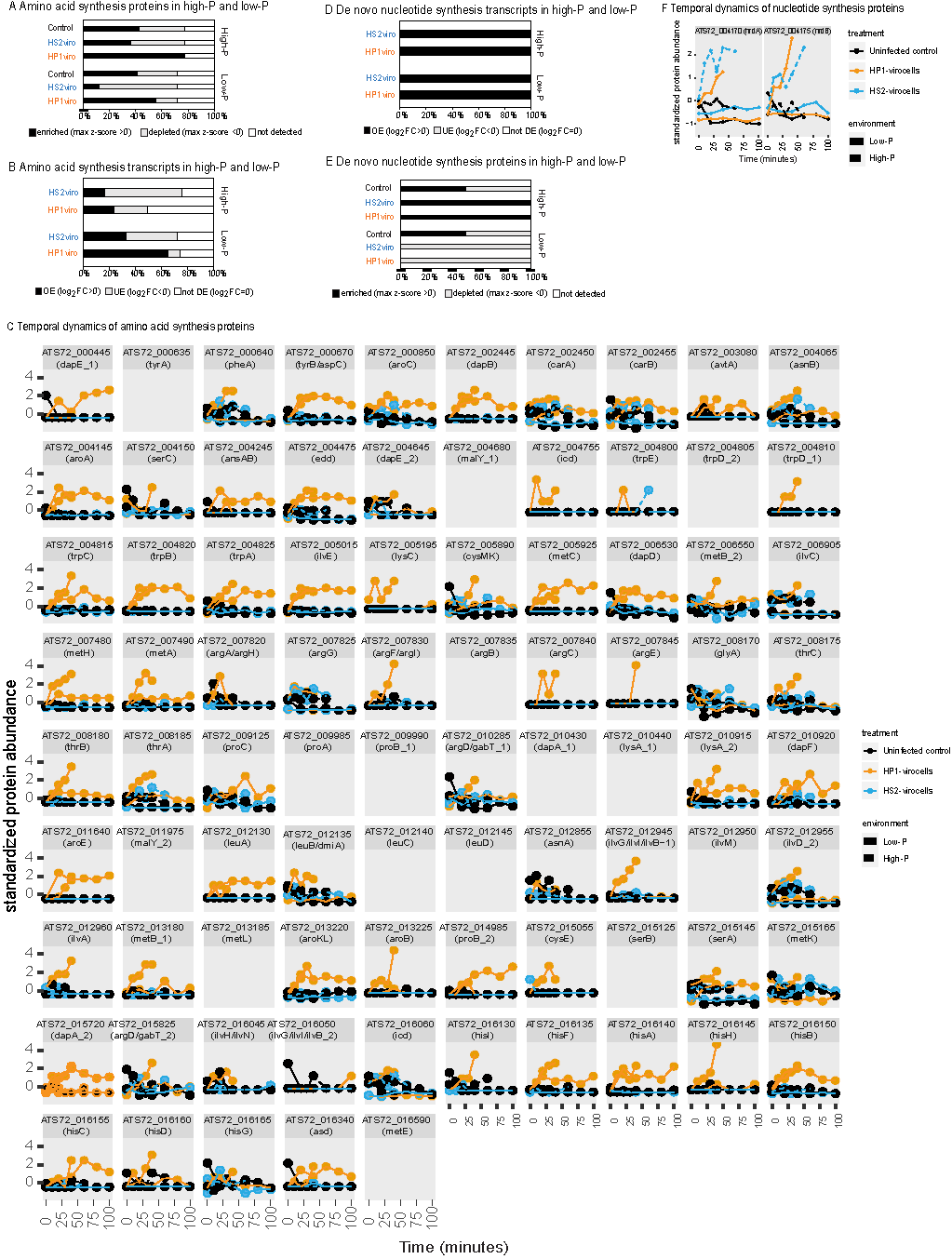


## Figure S3: Amino acid and nucleotide synthesis. A) Bar plot representing the fraction of amino acid synthesis proteins that are enriched (z-score>0), depleted (z-score<0), or not detected, in every treatment and environment, obtained from proteomics. B) Bar plot representing the fraction of amino acid synthesis genes over-expressed (OE), under-expressed (UE), or not differentially expressed (not DE) in the virocells relative to uninfected control cells in the respective environment, obtained from transcriptomics. C) Temporal dynamics of the amino acid synthesis proteins, plotted as standardized abundances (average z-score for three biological replicates) in high-P (dashed lines) and low-P (solid lines) media for the virocells and uninfected control cells, obtained from proteomics. D) Bar plot representing the fraction of ribonucleoside-diphosphate reductase (*nrd*) genes over-expressed (OE), under-expressed (UE), or not differentially expressed (not DE) in the virocells relative to uninfected control cells in the respective environment, obtained from transcriptomics. F) Bar plot representing the fraction of Nrd proteins that are enriched (z-score>0), depleted (z-score<0), or not detected, in every treatment and environment, obtained from proteomics.

##
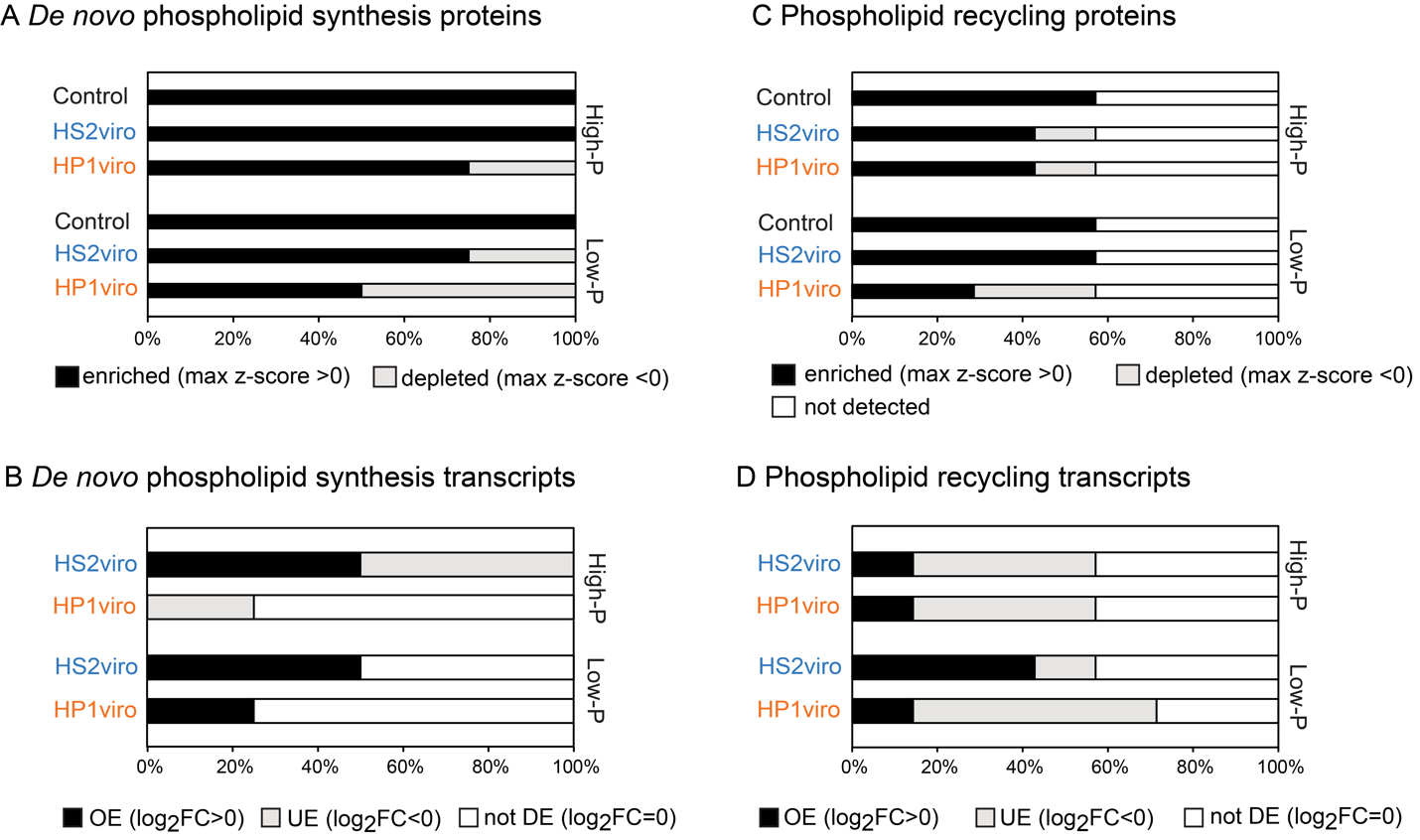


**Figure S4: Phospholipid synthesis. A)** Heatmap of the relative protein abundance of the phospholipid synthesis and recycling genes in both high-P and low-P conditions for uninfected control cells and both virocells, obtained from proteomics. Represented are z-scores. Proteins are considered enriched if z-score>0 and depleted if z-score<0. Dark gray denotes a protein not detected in our dataset. **B)** Heatmap of expression of the phospholipid synthesis and recycling genes in both high-P and low-P conditions for the virocells relative to uninfected control cells in each respective condition and time point, obtained from transcriptomics. Light gray denotes genes that are not differentially expressed (not DE). “OE” is over-expressed (log_2_FC>0), “UE” is under-expressed (log_2_FC<0).


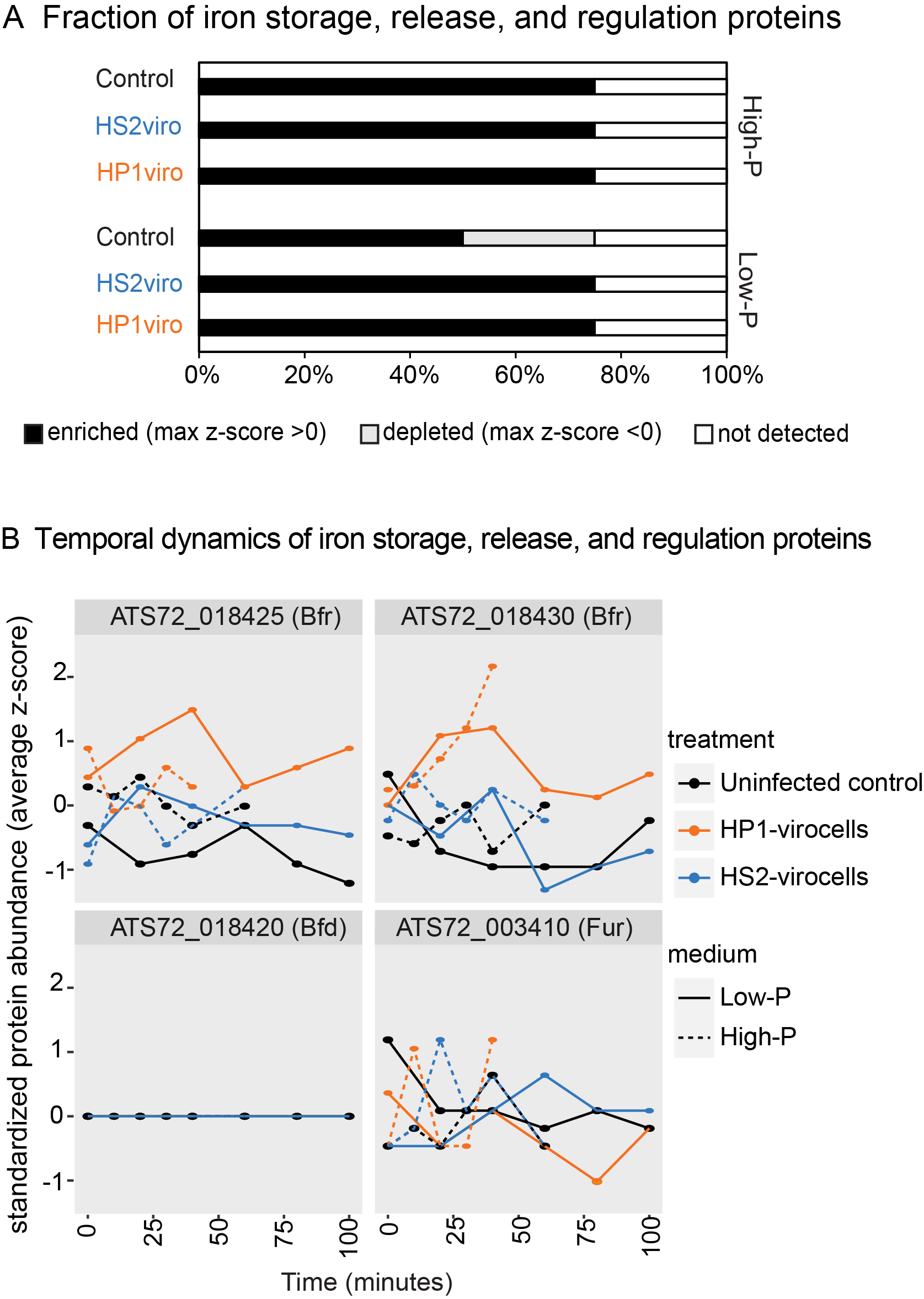


## Figure S5: Iron metabolism proteins. A) Bar plot representing the fraction of iron metabolism proteins that are enriched (z-score>0), depleted (z-score<0), or not detected, in every treatment and condition, obtained from proteomics. B) Temporal dynamics of the iron storage (Bfr), release (Bfd), and regulation (Fur) proteins, plotted as standardized abundances (average z-score for three biological replicates) in high-P (dashed lines) and low-P (solid lines) conditions for the virocells and uninfected control cells, obtained from proteomics.

##

## **Table S1. Results from the linear mixed effects model of amino acids from intracellular metabolomics data between conditions and infection types at the final time point.** All comparisons are made using all the 17 amino acids identified in the dataset from biological triplicate samples across all time points. The *P* values indicate if a variable significantly affects the response variable after controlling for other variables. The relative importance of the predictors can be inferred from the extra sum of squares explained values (Chi-squared). Degrees-of-freedom method: Kenward-Roger; *P* value adjustment: Turkey method for comparing a family of 3 estimates.

| **Marginal ANOVA** |  |  |
| --- | --- | --- |
| *Variable* | *Chi-Squared* | *P Value* |
| Infection type | 66 | **<0.05** |
| Media type | 2779 | **<0.05** |
| Time | 86 | **<0.05** |
| Interaction: infect *time | 75 | **<0.05** |
| Interaction: media* time | 407 | **<0.05** |
| **Contrasts** |  |  |
| *Host type* | *Mean difference*  *(high-P minus low-P, mean-centered intensity of metabolite)* | *P value* |
| Uninfected control | 1673899 | **<0.05** |
| HP1-virocell | 1889909 | **<0.05** |
| HS2-virocell | 679376 | **<0.05** |
| *Host differences* | *Mean difference*  *(high-P, mean-centered intensity of metabolite)* | *P value* |
| HP1-virocell - HS2-virocell | 747087 | **<0.05** |
| HP1-virocell - Control | 17232 | >0.05 |
| HS2-virocell - Control | -729765 | **<0.05** |
| *Host differences* | *Mean difference*  *(low-P, mean-centered intensity of metabolite)* | *P value* |
| HP1-virocell - HS2-virocell | -463446 | **<0.05** |
| HP1-virocell - Control | -198688 | **<0.05** |
| HS2-virocell - Control | 264758 | **<0.05** |

**Table S2.** **Results from the linear mixed effects model of the saturated lipids in the lipidomics data (mean-centered, log_2_-fold change of the intensity values).** The *P* values indicate if a variable significantly affects the response variable after controlling for other variables. The relative importance of the predictors can be inferred from the extra sum of squares explained values (Chi-squared). Degrees-of-freedom method: Kenward-Roger; *P* value adjustment: Turkey method for comparing a family of 3 estimates.

| **Marginal ANOVA** |  |  |
| --- | --- | --- |
| *Variable* | *Chi-Squared* | *P Value* |
| Infection type | 27.2355 | **<0.05** |
| Media type | 135.8721 | **<0.05** |
| Time | 0.0328 | >0.05 |
| Interaction: infect *time | 10.3372 | **<0.05** |
| Interaction: media* time | 2.2801 | >0.05 |
| **Contrasts** |  |  |
| *Host type* | *Mean difference*  *(high-P minus low-P, log2FC)* | *P value* |
| Uninfected control | 1.08 | **<0.05** |
| HP1-virocell | 1.50 | **<0.05** |
| HS2-virocell | 1.32 | **<0.05** |
| *Host differences* | *Mean difference*  *(high-P, log2FC)* | *P value* |
| Control – HP1-virocell | 0.423 | >0.05 |
| Control – HS2-virocell | 0.105 | >0.05 |
| HP1-virocell – HS2-virocell | -0.318 | >0.05 |
| *Host differences* | *Mean difference*  *(low-P, log2FC)* | *P value* |
| Control – HP1-virocell | 0.834 | **<0.05** |
| Control – HS2-virocell | 0.337 | >0.05 |
| HP1-virocell – HS2-virocell | -0.497 | **<0.05** |

**Table S3.** **Results from the linear mixed effects model of the unsaturated lipids in the lipidomics data (mean-centered, log2-fold change of the intensity values).** The *P* values indicate if a variable significantly affects the response variable after controlling for other variables. The relative importance of the predictors can be inferred from the extra sum of squares explained values (Chi-squared). Degrees-of-freedom method: Kenward-Roger; *P* value adjustment: Turkey method for comparing a family of 3 estimates.

| **Marginal ANOVA** |  |  |
| --- | --- | --- |
| *Variable* | *Chi-Squared* | *P Value* |
| Infection type | 1.7827 | >0.05 |
| Media type | 8.8936 | **<0.05** |
| Time | 0.0021 | >0.05 |
| Interaction: infect *time | 0.6766 | >0.05 |
| Interaction: media* time | 0.1492 | >0.05 |
| **Contrasts** |  |  |
| *Host type* | *Mean difference*  *(high-P minus low-P, log2FC)* | *P value* |
| Uninfected control | -0.139 | >0.05 |
| HP1-virocell | -0.192 | >0.05 |
| HS2-virocell | -0.169 | >0.05 |
| *Host differences* | *Mean difference*  *(high-P, log2FC)* | *P value* |
| Control – HP1-virocell | -0.0544 | >0.05 |
| Control – HS2-virocell | -0.0135 | >0.05 |
| HP1-virocell – HS2-virocell | 0.0409 | >0.05 |
| *Host differences* | *Mean difference*  *(low-P, log2FC)* | *P value* |
| Control – HP1-virocell | -0.1073 | >0.05 |
| Control – HS2-virocell | -0.0433 | >0.05 |
| HP1-virocell – HS2-virocell | 0.0639 | >0.05 |

##

## **Table S4.** Results from the linear mixed effects model of the unsaturated lipids in the lipidomics data (mean-centered, log2-fold change of the intensity values). All comparisons are made from biological triplicate samples across all time points and evaluated using a Wilcoxon test with *P* value adjustments for multiple pairwise comparisons were made using the Benjamini & Hochberg method. Classes of exometabolites are assigned using the countCompoundTypes function from the FT-ICR processing package by K. Todd Brown (<https://github.com/ktoddbrown/FTICR_Processing)>).

| variable | y | group1 | group2 | p | p.adj | p.adj.signif |
| --- | --- | --- | --- | --- | --- | --- |
| Unsaturated Hydrocarbon | C | P.poor | P.rich | 0.65100000 | 0.71834482759 | ns |
| Polyphenol | C | P.poor | P.rich | 0.31600000 | 0.52218181818 | ns |
| Carboxylated/Oxygen Rich | C | P.poor | P.rich | 0.12400000 | 0.23341176471 | ns |
| Unsaturated Hydrocarbon | HP1 | P.poor | P.rich | 0.00280000 | 0.01120000000 | * |
| Polyphenol | HP1 | P.poor | P.rich | 0.00028800 | 0.00131657143 | ** |
| Carboxylated/Oxygen Rich | HP1 | P.poor | P.rich | 0.00006660 | 0.00035520000 | *** |
| Unsaturated Hydrocarbon | HS2 | P.poor | P.rich | 0.48700000 | 0.59938461538 | ns |
| Polyphenol | HS2 | P.poor | P.rich | 0.04390000 | 0.11706666667 | ns |
| Carboxylated/Oxygen Rich | HS2 | P.poor | P.rich | 0.34700000 | 0.52218181818 | ns |
| Unsaturated Hydrocarbon | All cells | P.poor | P.rich | <0.0001 | 0.07106 | ns |
| Polyphenol | All cells | P.poor | P.rich | 0.00067 | 0.00135 | ** |
| Carboxylated/Oxygen Rich | All cells | P.poor | P.rich | 0.00005 | 0.00014 | *** |
| Unsaturated Hydrocarbon | Low-P | C | HP1 | 0.190 | 0.4342857 | ns |
| Unsaturated Hydrocarbon | Low-P | C | HS2 | 0.887 | 0.9461333 | ns |
| Unsaturated Hydrocarbon | Low-P | HP1 | HS2 | 0.079 | 0.3729231 | ns |
| Polyphenol | Low-P | C | HP1 | 0.051 | 0.3497143 | ns |
| Polyphenol | Low-P | C | HS2 | 0.887 | 0.9461333 | ns |
| Polyphenol | Low-P | HP1 | HS2 | 0.022 | 0.2640000 | ns |
| Carboxylated/Oxygen Rich | Low-P | C | HP1 | 0.151 | 0.4026667 | ns |
| Carboxylated/Oxygen Rich | Low-P | C | HS2 | 0.273 | 0.5241600 | ns |
| Carboxylated/Oxygen Rich | Low-P | HP1 | HS2 | 0.525 | 0.6953514 | ns |
| Unsaturated Hydrocarbon | High-P | C | HP1 | 0.695 | 0.8778947 | ns |
| Unsaturated Hydrocarbon | High-P | C | HS2 | 0.898 | 0.9578667 | ns |
| Unsaturated Hydrocarbon | High-P | HP1 | HS2 | 0.651 | 0.8445405 | ns |
| Polyphenol | High-P | C | HP1 | 0.786 | 0.9201951 | ns |
| Polyphenol | High-P | C | HS2 | 0.270 | 0.6168889 | ns |
| Polyphenol | High-P | HP1 | HS2 | 0.880 | 0.9578667 | ns |
| Carboxylated/Oxygen Rich | High-P | C | HP1 | 0.525 | 0.7411765 | ns |
| Carboxylated/Oxygen Rich | High-P | C | HS2 | 0.171 | 0.5862857 | ns |
| Carboxylated/Oxygen Rich | High-P | HP1 | HS2 | 0.044 | 0.2784000 | ns |
